# A broad autism phenotype expressed in facial morphology

**DOI:** 10.1038/s41398-020-0695-z

**Published:** 2020-01-16

**Authors:** Diana Weiting Tan, Murray T. Maybery, Syed Zulqarnain Gilani, Gail A. Alvares, Ajmal Mian, David Suter, Andrew J. O. Whitehouse

**Affiliations:** 1 School of Psychological Science, University of Western Australia, Perth, Australia; 2 Telethon Kids Institute, University of Western Australia, Perth, Australia; 3 School of Computer Science and Software Engineering, University of Western Australia, Perth, Australia; 4 School of Sciences, Edith Cowan University, Perth, Australia

**Keywords:** Psychology, Physiology

## Abstract

Autism spectrum disorder is a heritable neurodevelopmental condition diagnosed based on social and communication differences. There is strong evidence that cognitive and behavioural changes associated with clinical autism aggregate with biological relatives but in milder form, commonly referred to as the ‘broad autism phenotype’. The present study builds on our previous findings of increased facial masculinity in autistic children (Sci. Rep., 7:9348, 2017) by examining whether facial masculinity represents as a broad autism phenotype in 55 non-autistic siblings (25 girls) of autistic children. Using 3D facial photogrammetry and age-matched control groups of children without a family history of ASD, we found that facial features of male siblings were more masculine than those of male controls (*n* *=* 69; *p* *<* 0.001, *d* = 0.81 [0.36, 1.26]). Facial features of female siblings were also more masculine than the features of female controls (*n* *=* 60; *p* *=* 0.005, *d* = 0.63 [0.16, 1.10]). Overall, we demonstrated for males and females that facial masculinity in non-autistic siblings is increased compared to same-sex comparison groups. These data provide the first evidence for a broad autism phenotype expressed in a physical characteristic, which has wider implications for our understanding of the interplay between physical and cognitive development in humans.

## Introduction

Among the neurodevelopmental conditions, autism spectrum disorder (ASD) is considered to be the most heritable^1^, with an increasing probability of diagnosis as a function of genetic relatedness. While ASD is diagnosed in approximately 1% of the population^2^, the probability of a second diagnosis in a family increases to around 13% in full siblings and dizygotic twins, and to around 59% in monozygotic twins^3^. Among non-autistic first-degree relatives of autistic children, subclinical autistic-like characteristics, often referred to as the ‘broad autism phenotype’, are also commonly reported. For instance, compared to individuals with no known family history of ASD, non-autistic parents and siblings of autistic individuals have exhibited higher levels of autistic-like traits^4^, poorer language abilities^5^, and greater social-communication difficulties^6^.

ASD is three times more frequently diagnosed in males than in females^7^. The ‘extreme male brain’ theory hypothesises that the male preponderance in ASD may be associated with heightened exposure to testosterone *in utero*^8^. Testosterone is a sex steroid that is critical for male virilisation, hence its production is more pronounced in pregnancies with a male fetus than those with a female^9^. Testosterone crosses the blood-brain barrier and can influence fetal brain development during pregnancy. Prior studies investigating the association between prenatal testosterone exposure and autistic-like characteristics have typically focused on neurotypical individuals from the general population, in part, due to the challenge of collecting biological specimens for the analyses of prenatal testosterone. Several studies have reported higher levels of prenatal testosterone derived from amniotic fluid associated with higher levels of autistic traits^10,11^ and poorer language outcomes^12^. However, other studies of either prenatal or perinatal testosterone have not observed this type of association^13,14^. To date, only one study has investigated levels of prenatal testosterone in relation to ASD outcome in boys by linking national health and psychiatric records in Denmark^15^. A group of sex steroids involved in the biosynthesis of androgen was collectively elevated in the amniotic fluid samples of boys who were later diagnosed with ASD compared to boys of typical development. Overall, existing evidence for the association between prenatal testosterone and autistic-like characteristics is mixed in the general population and shows some promise in the clinical population.

During the earliest stage of fetal development, the brain and the face unfold from the neural crest in close coordination^16^. This has led to another line of research focusing on the relationship between testosterone exposure during pregnancy and its effects on facial morphology. Whitehouse et al.^17^ found that more masculinised facial structures in young men and women were related to increased levels of testosterone measured from their umbilical cords collected at birth. Given the possible links between prenatal testosterone and the masculinisation of the brain and face during fetal development, investigations motivated by the extreme male brain theory have been extended to studying facial masculinisation in children with ASD. Although facial structures are known to be most sexually dimorphic after the onset of puberty^18^, recent advances in three-dimensional (3D) photogrammetry have allowed for facial masculinity to be defined in prepubescent infants and children^19–21^.

Using 3D facial scans, we reported the first evidence of a masculinised facial structure in autistic children, based on a two-phase investigation^21^. In the first phase, a gender classification algorithm was developed to select and combine a set of facial distances measured between landmarks that optimally classified male and female faces in a sample of typically developing prepubescent children. This algorithm was used in the second phase whereby a ‘gender score’ was computed for 3D facial scans of 74 autistic children (20 girls) and 114 non-autistic children (60 girls; a detailed description is provided in Tan et al.^21^). We found that relative to non-autistic children, autistic children included in this study presented substantially more masculine gender scores and facial distances.

Facial features are highly heritable. Employing a monozygotic-dizygotic twin design, Djordjevic et al.^22^ reported that genetic factors accounted for more than 70% of the overall variation in facial features. In another study, Lee and colleagues^23^ examined whether facial masculinity was influenced by genetic factors amongst pairs of monozygotic and dizygotic twin adolescents. For each subject, a facial masculinity score was established from facial features measured from 18 landmarks placed on two-dimensional facial photographs, which had contributed to the overall gender classification using discriminant function analysis. The authors reported that genetic factors explained 46 and 48% of the variability in facial masculinity in males and females, respectively. Therefore, it was concluded that facial masculinity is a heritable trait with strong genetic influences.

While there is strong evidence for the heritability of facial masculinity as well as ASD, it is unknown whether the masculinised facial structures previously observed in autistic children^21^ are also present in non-autistic full siblings of autistic children. At present, only two studies have examined the facial morphology of typically developing siblings. Hammond et al.^24^ examined facial asymmetry as a potential index of brain dysmorphogenesis in children with ASD, using their non-autistic siblings and unrelated children without a family history of ASD as comparison groups. Children with ASD presented with the most pronounced facial asymmetry while their siblings and the unrelated comparison group were equivalent in asymmetry. Hammond et al.’s findings were replicated by Boutrus et al.^25^ who also observed more asymmetric facial morphology among autistic children compared to non-autistic siblings and unrelated children. As in the Hammond et al. study, facial asymmetry in the sibling and control groups did not differ. By including non-autistic sibling samples, the authors were able to conclude that facial asymmetry may be specifically associated with an etiological mechanism specific to ASD, rather than a genetic liability within the family.

The current study investigated facial masculinity in non-autistic siblings of autistic children and in children without a family history of ASD. There were two aims in this study. First, we examined the generalisation of the Tan et al. gender classification algorithm by using 3D facial scans of a new sample of neurotypical children drawn from the general population. Second, the degree of facial masculinity was compared between non-autistic siblings of autistic children and neurotypical children with no known family history of ASD. Given previous evidence of (i) facial masculinisation in children with ASD, (ii) the presence of a broad autism phenotype among non-autistic relatives of autistic individuals, and (iii) the heritability of facial masculinity, we hypothesised that facial masculinity would be more pronounced for the male and female sibling groups compared to their same-sex comparison groups.

## Method

### Participants

A total of 209 children (109 boys: mean age = 7.56 years, SD = 2.44, range = 2.68–12.56; 100 girls: mean age = 7.44 years, SD = 2.42, range = 2.95–12.29) with no known family history of ASD were recruited from the general population at community events in Perth, Western Australia. Samples were restricted to those of Caucasian descent to minimise effects of ethnic variability. Of these, 40 boys (mean age = 7.85 years, SD = 2.30, range = 3.17–12.11) and 40 girls (mean age = 7.51 years, SD = 2.40, range = 2.95–12.29) were selected based on their ages to evaluate the generalisation of the gender classification algorithm reported in Tan et al.^21^. These boys and girls were matched on age (*p* = 0.52); ages were also not significantly different from the ages of children included in the algorithm training phase in Tan et al.^21^ (*p* = 0.99 for boys and *p* = 0.45 for girls).

The remaining 69 boys (mean age = 7.39 years, SD = 2.51, range = 2.95–12.56) and 60 girls (mean age = 7.40 years, SD = 2.42, range = 3.04–12.28) formed same-sex comparison group for 30 non-autistic male siblings (mean age = 7.54 years, SD = 2.65, range = 2.91–12.59) and 25 non-autistic female siblings (mean age = 7.39 years, SD = 2.48, range = 2.83–11.91) of autistic children recruited from the Telethon Kids Institute, Perth, Western Australia. We conducted a power analysis using G*Power^26^. Based on effect sizes reported in Tan et al.^21^, Cohen’s *f* of 0.42 and 0.82 were used to determine the minimum sample size required to detect effects of ASD diagnosis on facial masculinity in boys and girls respectively. Analyses suggest that the minimum sample size required to achieve 90% power was 62 for boys and 18 for girls. Hence, the current sample size is deemed as adequate. None of these participants are siblings of the autistic probands included in Tan et al.^21^ and all siblings in the present study were unrelated to one another. Parents reported no history of facial trauma or known syndromic disorders for all of the participants. Parents also provided written informed consent and ethics approval was sought and granted by the Human Research Ethics Committee in the University of Western Australia (RA/4/1/5657).

### Facial photography

3D facial images were obtained using a 3dMDface system (3dMD, Atlanta, GA, USA). From two stereo camera viewpoints placed on either side of each child, the 3dMDface system projects random infrared lights on the child’s face to establish correspondence between images taken from either viewpoint, thus creating a 3D facial model with high precision (error less than 2 mm) and high reliability^27^. During the imaging process, each child sat in front of the 3dMDface system, attempted a neutral facial expression, and kept their lips closed.

### Gender classification and scoring algorithms

**Fig. 1:**
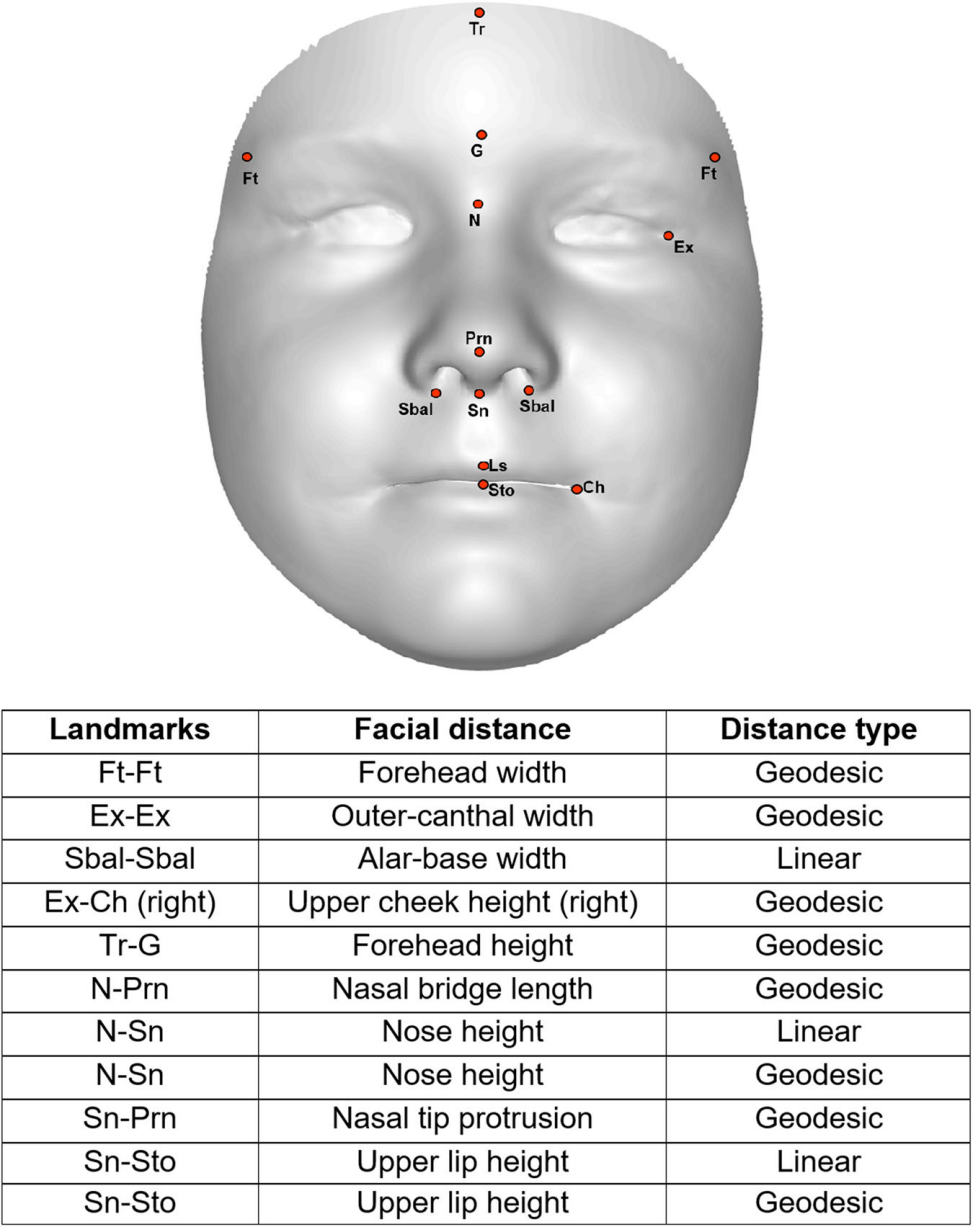
A composite facial image annotated with 13 facial landmarks and a summary of the landmark names, distances, and distance types measured in the current study. Facial landmarks were based on the definitions described in Farkas^39^.

In the present study, we examined the generalisation of the gender classification algorithm trained and established in Tan et al.^21^. The steps involved in the algorithm are summarised in the Supplementary Material (see Figure S1). For the current study, 13 landmarks were manually placed on each 3D facial image, and the three linear and eight geodesic distances used in the classification algorithm were derived (see Fig. 1 for landmark locations and definitions). These 11 distances were then entered into the Linear Discriminant Analysis (LDA) classifier to determine how well the algorithm could classify the new groups of 40 boys and 40 girls according to their sex, respectively.

Following this, a three-step gender scoring algorithm described in Tan et al.^21^ was employed to compute an overall facial masculinity score for each of the children in the sibling and comparison groups. First, 13 landmarks were annotated on the 3D facial scan of each child (hereafter the ‘test face’) and the 11 distances described in Fig. 1 were measured. Next, these facial distances were projected in the LDA space established in the Tan et al. study for representing the two gender classes. Finally, the deviation between the test face and the means of the male and female classes was used in the calculation of a gender score. For the gender score in this study, a score of 0 represents extreme femininity and 20 represents extreme masculinity. The gender score will be referred to as the ‘facial masculinity score’ from here on. In addition, as variations in body mass index and head size may influence measurements of facial distances, facial areas were calculated by adding the triangular areas connected between the points in the 3D space.

### Statistical analyses

All statistical analyses were conducted using RStudio^28,29^. Welch’s *t* tests were conducted to compare the facial areas and each of the 11 facial distances (previously found to optimally contribute to the gender classification accuracy in Tan et al.^21^) between the 40 boys and 40 girls included in the validation of the gender classification algorithm. Welch’s *t* tests were also employed to compare the sibling and comparison groups on their facial areas, facial masculinity scores, and facial distances that have been found to be sexually dimorphic. For any variable that violated the assumptions of parametric tests, Wilcoxon signed-rank test was used. An alpha level of .05 and effect sizes were considered in determining statistical significance.

## Results

### Generalisation of the gender classification algorithm reported in Tan et al.^21^

Based on the 11 facial distances, the gender classification algorithm correctly classified the sex of the 40 boys and 40 girls with an accuracy of 95.4% for boys and 96.0% for girls. Facial areas were not statistically significantly different between boys and girls (*p* = 0.12, *d* = 0.35). Seven of the 11 features were significantly different between boys and girls (see Table 1). Of these, six features (linear alar-base width, linear upper lip height, geodesic outer-canthal width, geodesic forehead width, geodesic nose height, and geodesic upper lip height) were larger in boys than in girls (largest *p* = 0.01, *d* = 0.56). Consistent with Tan et al.^21^, geodesic forehead height was larger in girls than in boys (*p* < 0.001, *d* = 1.17). The high accuracy of gender classification and replication of sex differences on individual features supports the generalisation of the Tan et al. gender classification algorithm.

**Table 1:** Descriptive and null hypothesis testing statistics for facial distances of typically developing boys and girls.

		Boys (*n* = 40)		Girls (*n* = 40)		
Facial variables	Model Weight	*M*	SD	*M*	SD	Test statistics
Facial area (mm^2^)^a^	–	28 373	5 580	26 674	4 903	*W* = 961, *p* = 0.12, *d* = 0.35 [–0.09, 0.79]
*Linear distances (mm)*						
Alar-base width^bc^	0.38	15.0	1.52	13.9	1.73	*t*(76.6) = 2.88, *p* = 0.005, *d* = 0.64 [0.19, 1.10]
Nose height^b^	1.54	38.7	3.28	37.9	4.00	*t*(75.1) = 0.96, *p* = 0.34, *d* *=* 0.21 [−0.23, 0.66]
Upper lip height^bc^	0.92	22.8	2.41	20.4	2.35	*t*(78.0) = 4.37, *p* < 0.001, *d* = 0.98 [0.51, 1.45]
*Geodesic distances (mm)*						
Outer-canthal width^bc^	0.39	100.6	8.07	94.6	7.11	*t*(76.8) = 3.52, *p* = 0.0007, *d* = 0.79 [0.33, 1.25]
Forehead height^bc^	1.55	50.4	6.71	57.6	5.39	*t*(74.5) = 5.24, *p* < 0.001, *d* = 1.17 [0.69, 1.65]
Forehead width^c^	0.52	147.6	9.38	141.6	12.1	*t*(73.5) = 2.51, *p* = 0.01, *d* = 0.56 [0.11, 1.01]
Right upper cheek height	1.13	66.4	5.10	65.2	4.14	*t*(74.8) = 1.17, *p* = 0.25, *d* = 0.26 [−0.19, 0.71]
Nasal tip protrusion^a^	1.31	14.8	2.35	14.3	2.72	*W* = 954, *p* = 0.14, *d* = 0.38 [−0.12, 0.76]
Nose height^bc^	5.42	49.4	3.93	45.7	4.92	*t*(74.3) = 3.66, *p* = 0.0005, *d* = 0.82 [0.35, 1.28]
Upper lip height^c^	0.54	25.5	3.33	22.4	3.34	*t*(78.0) = 4.05, *p* = 0.0001, *d* = 0.90 [0.44, 1.37]
Nasal bright length	3.59	33.6	3.28	32.5	4.33	*t*(72.6) = 1.24, *p* = 0.22, *d* = 0.28 [–0.17, 0.73]

^a^*M* and SD replaced with median and interquartile range, respectively

^b^Statistically significantly different between boys and girls in Tan et al.^21^

^c^Statistically significantly different in the present study

### Comparison between sibling and control groups

For each sex, we compared the sibling and control groups on their facial areas, overall facial masculinity scores, as well as on the eight facial distances that were statistically significantly different between typically developing boys and girls either in Tan et al.^21^ or in the current study (i.e., features denoted by b and/or c in Table 1).

Descriptive and test statistics for boys are presented in Table 2 and those for girls are in Table 3. For both sexes, facial areas did not differ between the sibling group and their same-sex counterparts (boys: *p* = 0.34, *d* *=* 0.22; girls: *p* = 0.36, *d* *=* 0.24). For boys, we found a strong masculinised shift in the sibling group relative to their male counterparts in their overall facial masculinity scores. In terms of their facial distances, there were strong masculinised effects across all distances (smallest *d* = 0.63) except linear nose height and geodesic forehead width. For girls, there was also a moderately strong masculinised shift among siblings compared to female controls in their overall facial masculinity scores. As for their facial distances, there were moderately strong masculinised effects in two of the eight distances, which were linear alar-base width and geodesic upper lip height (smaller *d* = 0.82). The remaining distances show either weak evidence of masculinisation (linear nose height and upper lip height, and geodesic forehead width) or no masculinisation.

**Table 2:** Descriptive and null hypothesis testing statistics for the facial masculinity score and distances for male siblings and age-matched male controls.

	Siblings (*n* = 30)		Controls (*n* = 69)		
Facial variables	*M*	SD	*M*	SD	Test statistics
Facial area (mm^2^)	27 446	4 331	26 578	3 720	*t*(48.5) = 0.95, *p* = 0.34, *d* = 0.22 [–0.66, 0.21]
Masculinity score^ab^	14.0	2.81	11.6	3.19	*t*(62.3) = 3.89, *p* = 0.0002, *d* = 0.81 [0.36, 1.26]
*Linear distances (mm)*					
Alar-base width^abc^	15.6	2.07	14.2	2.06	*W* = 570, *p* = 0.0004, *d* = 0.77 [0.33, 1.21]
Nose height^a^	38.0	3.60	36.9	4.68	*t*(71.0) = 1.22, *p* = 0.23, *d* = 0.24 [–0.20, 0.68]
Upper lip height^ab^	24.2	2.64	21.2	2.28	*t*(48.8) = 5.32, *p* < 0.001, *d* = 1.23 [0.76, 1.70]
*Geodesic distances (mm)*					
Outer canthal width^ab^	100.7	7.19	95.0	8.03	*t*(61.3) = 3.50, *p* = 0.0009, *d* = 0.73 [0.29, 1.18]
Forehead height^b^	51.6	9.03	57.0	8.59	*t*(52.8) = 2.82, *p* = 0.007, *d* = 0.63 [0.19, 1.07]
Forehead width^b^	147.9	10.9	142.7	12.1	*t*(61.1) = 2.10, *p* = 0.04, *d* = 0.44 [0.002, 0.88]
Nose height^ab^	49.3	4.68	43.9	5.78	*t*(67.6) = 4.83, *p* < 0.001, d = 0.97 [0.52, 1.43]
Upper lip height^b^	26.7	3.21	23.5	3.28	*t*(56.3) = 4.41, *p* < 0.001, *d* = 0.96 [0.50, 1.41]

^a^Statistically significantly different between autistic and non-autistic boys in Tan et al.^21^

^b^Statistically significantly different in the present study

^c^M and SD replaced with median and interquartile range, respectively

**Table 3:** Descriptive and null hypothesis testing statistics for the facial masculinity score and distances for female siblings and age-matched female controls.

Facial variables	Siblings (*n* = 25)		Controls (*n* = 60)		
	*M*	SD	*M*	SD	Test statistics
Facial area (mm^2^)	26 865	4 053	26 029	3 273	*t*(37.7) = 0.91, *p* = 0.36, *d* = 0.24 [–0.24, 0.71]
Masculinity score^abc^	5.17	3.27	3.67	2.63	*W* = 459, *p* = 0.005, *d* = 0.63 [0.15, 1.10]
*Linear distances (mm)*					
Alar-base width^bc^	14.0	1.75	12.7	1.57	*t*(40.8) = 3.30, *p* = 0.002, *d* = 0.82 [0.33, 1.31]
Nose height^bc^	37.6	3.56	35.6	3.85	*t*(48.4) = 2.24, *p* = 0.03, *d* = 0.54 [0.04, 1.00]
Upper lip height^ab^	21.6	2.17	21.0	2.21	*W* = 560, p = 0.07, *d* = 0.41 [–0.06, 0.88]
*Geodesic distances (mm)*					
Outer canthal width^b^	95.4	6.59	93.6	7.59	*t*(51.5) = 1.12, *p* = 0.27, *d* = 0.25 [−0.72, 0.22]
Forehead height	53.8	7.86	55.8	6.57	*t*(38.7) = 1.10, *p* = 0.28, *d* = 0.28 [−0.19, 0.76]
Forehead width^c^	143.5	9.01	137.2	9.81	*t*(48.7) = 2.86, *p* = 0.006, *d* = 0.66 [0.17, 1.14]
Nose height^b^	45.2	3.82	44.1	4.66	*t*(54.5) = 1.14, *p* = 0.26, *d* = −0.25 [−0.73, 0.22]
Upper lip height^ac^	25.1	4.40	22.8	2.47	*W* = 351, *p* = 0 .0001, *d* = 0.93 [0.44, 1.41]

^a^*M* and SD replaced with median and interquartile range, respectively

^b^Statistically significantly different between autistic and non-autistic girls in Tan et al.^21^

^c^Statistically significantly different in the present study

### Comparison between autistic probands, siblings, and control groups

**Fig. 2:**
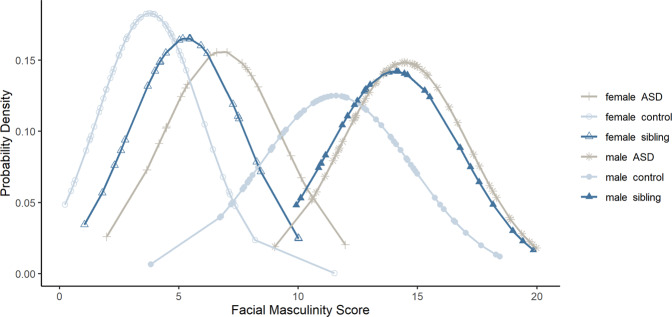
Probability density function depicting the distribution of facial masculinity scores for each group. Scores of autistic females, female siblings, and female controls are represented by crosses, unfilled triangles, and unfilled circles, respectively. Scores of autistic males, male siblings, and male controls are represented by stars, filled triangles, and filled circles, respectively.

We conducted an unplanned analysis to compare facial masculinity scores of autistic probands included in Tan et al.^21^ with the sibling and control samples included in the present study. For boys, one-way ANOVA revealed a significant effect of group, *F*(2,150) = 16.8, *p* < 0.001. Post hoc comparisons using the Tukey HSD test indicated that autistic boys (*M* = 14.5, SD = 2.69) and male siblings presented higher masculinity scores than male controls (all *p*s < .001). However, masculinity score did not differ between the autistic boys and sibling boys. Similarly, there was a significant effect of group in girls, *F*(2,102) = 14.0, *p* < 0.001, with autistic girls (*M* = 6.81, SD = 2.56) and female siblings showing higher masculinity scores than female controls (*p* < 0.001 and *p* = 0.02, respectively). Facial masculinity scores were marginally higher in autistic girls than in sibling girls (*p* = 0.08). The distributions of the masculinity scores for the six groups of children are presented in Fig. 2, where a rightward shift towards extreme masculinity is evident for the sibling and autistic samples.

## Discussion

The current study provided evidence supporting the hypothesis that facial masculinity would be more pronounced in typically developing boys and girls with a family history of ASD compared to those without. These findings extend our previous work which reported increased facial masculinity among children diagnosed with ASD^21^. Overall, we provide evidence for a broad autism phenotype expressed in facial masculinity among non-autistic siblings of autistic children.

There appears to be stronger evidence of facial masculinisation among male siblings compared to female siblings. In boys, seven of the eight facial distances differed in the masculinised direction in the sibling group relative to their male control counterparts, with each effect large in magnitude. Of the seven distances, five were also found to be more masculinised in autistic children in Tan et al.^21^. On the other hand, in girls, only four of the eight distances showed some evidence of hypermasculinity in the sibling group relative to their female control counterparts, with each effect ranging from medium to large in magnitude. Of these four distances, two are consistent with the previous comparisons between autistic female probands and non-autistic female controls^21^. Additionally, we examined the distributions of the facial masculinity scores of autistic probands included in Tan et al.^21^, and scores of the siblings and control groups included in the current study. Facial scores of the male probands and siblings showed a similar degree of masculinisation relative to male controls (i.e., male probands = male siblings > male controls) while facial scores of female groups were spread in a graded manner, with the scores of female siblings expressing an intermediate phenotype between probands and controls (i.e., female probands > female siblings > female controls).

These patterns of results directly mirror those of a recent study which examined levels of autistic traits measured using the child version of the Autism-spectrum Quotient (AQ-Child^30^) among three samples of children aged 4 to 11 years—male and female autistic probands, their non-autistic siblings, and children drawn from families with no history of ASD (controls)^4^. There was no difference in AQ-Child scores between male autistic probands and non-autistic siblings; both groups presented higher AQ-Child scores than male control children (i.e., male probands = male siblings > male controls). Among females, AQ-Child scores presented in the sibling group fell between the scores for the autistic probands and non-autistic controls (i.e., female probands > female siblings > female controls). Taken together, these findings are consistent with other studies which found that the broad autism phenotype tends to aggregate in male relatives more than in female relatives^31,32^. Thus, while the pattern of differences in facial masculinity reported in the present study warrants replication with larger samples, the results are consistent with facial masculinity signalling greater susceptibility to ASD.

This study has many strengths including a replication of the previously established gender classification algorithm, a novel extension of previous findings to non-autistic siblings of children with ASD, and the use of highly reliable and precise 3D photogrammetry. Additionally, because none of the siblings included in this study were related to the autistic probands included in Tan et al.^21^, the evidence of facial masculinisation observed in the current sibling sample is independent of the evidence of face masculinisation in autistic children reported by Tan et al.^21^. Nevertheless, the findings reported in the present study are subject to three limitations. First, we restricted our sample to children of Caucasian descent to limit the potential influence of variance in facial morphology as a function of ethnicity. Hence, it is unclear whether these findings would generalise to other ethnic populations. Second, the current study examined full siblings of autistic probands, therefore it is difficult to determine whether hypermasculinised facial structures in the sibling group arose from heritable genetic liability, shared maternal and/or paternal factors (e.g., maternal health^33^), or an interaction of these factors. Third, the current study included samples of children with a wide age range (2.83–12.59 years), and it is possible that the differences in facial masculinisation observed may be driven by larger differences among older children approaching pubertal age^18^. In the Tan et al. study, which included larger samples of children of a similar age range (3.01–12.52 years), it was possible to include an ‘age’ factor (i.e., ‘younger’ versus ‘older’ groups) in the analyses of group differences between autistic and non-autistic children. A main effect of age was reported in the Tan et al. study, but the interaction between ASD diagnosis and age group was not statistically significantly, indicating that the differences in facial masculinity between autistic and non-autistic children are uniform among younger and older children. While the limited size of the current study sample precluded the introduction of an age factor in the analyses, the Tan et al. study provides evidence that age may not exert a large effect on the differences observed.

The current study presents the first evidence for facial masculinity to express as a broad autism phenotype. This finding builds upon prior evidence linking prenatal testosterone exposure to postnatal facial masculinity^17^ and corroborates the ‘extreme male brain theory’ that ASD may be, in part, linked to elevated levels of testosterone *in utero*. More broadly, these data suggest that facial masculinity is a feature of ASD that is likely to be connected to genetic influences. Future research may investigate mechanism(s) that underlie prenatal brain and face developments that are associated with the developmental cascade leading to a diagnosis of ASD. One possible research avenue is an investigation of masculinity expressed in the faces of the biological parents of children with ASD. This will provide clues suggesting whether masculinised facial structures associated with ASD are influenced by maternal and/or paternal genetic inheritance. Second, as it is hypothesised that multiplex families (i.e., families with more than one autistic proband) possess a greater genetic liability than simplex families (i.e., families with one autistic proband)^34^, a study that compares facial masculinity expressed in members of multiplex versus simplex families may add to the current evidence that increased facial masculinity is associated with higher genetic liability associated with ASD. Third, while one study has shown that elevated levels of testosterone exposure during pregnancy are linked to facial masculinisation in adulthood, future studies could consider other early pregnancy factors that may influence the elevation of prenatal testosterone and the subsequent development of masculine features. One such factor is increased maternal weight which has been linked to higher levels of prenatal testosterone^35^ and to autistic traits^36^. Future research could also consider the potential etiological influence of nausea and vomiting during pregnancy which has been found to be related to increased symptom severity in ASD^37^. Maternal nausea and vomiting during pregnancy is thought to involve the dysregulation of several hormones including estrogens^38^ which may have feminising properties.

In conclusion, the present study found that both male and female non-autistic siblings of autistic children presented with more masculinised facial structures compared to their age- and sex-matched counterparts. To the best of our knowledge, these data provide the first evidence for a broad autism phenotype expressed in a physical characteristic, which has wider implications for our understanding of the interplay between physical and neurocognitive development.

## Supplementary information

Supplementary Material

## Data Availability

Data and R codes are publicly available at https://github.com/dianawtan/asd-sibs-faces.
