# Supplementary material for: A broad autism phenotype expressed in facial morphology
Source: Transl Psychiatry. 2020 Jan 16;10:7. doi: 10.1038/s41398-020-0695-z (PMC7026150; doi:10.1038/s41398-020-0695-z)

**Supplementary Material**

We reported a two-phase investigation in Tan et al. (2017) to examine the degree of facial masculinity in prepubescent children with and without autism spectrum disorder (ASD). We first determined facial features that characterise ‘masculinity’ in the first phase by using machine learning to select and combine a set of facial distances that optimally classified male and female faces in a sample of typically developing prepubescent children (48 boys: mean age = 7.86 years, SD = 3.10, range = 3.01–12.40; 53 girls: mean age = 7.93 years, SD = 2.77, range = 3.01–12.44). A gradient-based efficient feature selection algorithm and linear discriminant analysis classifier (collectively referred to as the ‘gender classification algorithm’) identified a combination of 11 facial features capable of classifying the boys and girls with an accuracy of 98.3% and 97.8%, respectively. The gender classification algorithm developed in this first phase enabled the computation of a ‘gender score’ for any ‘test’ face, representing its position along a male-female axis in a multi-dimensional feature space (see Figure S1).

In the present study, we examined the generalisation of the trained gender classification algorithm with 80 new test faces collected from 40 typically developing boys (mean age = 7.85 years, SD = 2.30, range = 3.17–12.11) and 40 typically developing girls (mean age = 7.51 years, SD = 2.40, range = 2.95–12.29) with no known family history of ASD. The 11 critical features were measured from 13 landmarks (see Figure 1) and entered into the algorithm. The gender classification algorithm classified the new ‘test’ faces with an accuracy of 95.4% for boys and 96.0% for girls.

**Discussion**

In this study, we demonstrated the generalisation of the gender classification algorithm reported in Tan et al. (2017). Using a new sample of 40 boys and 40 girls recruited from the general population, we found that classification accuracy remained highly accurate at 95.4% and 96.0% in boys and girls, respectively (c.f. Tan et al.: 98.3% and 97.8% in boys and girls, respectively). Furthermore, four of the six facial distances previously found to be statistically significantly larger in boys than in girls reported by Tan and colleagues were replicated in the current study (replicated distances denoted by ^ab^ in Table 1). Consistent with Tan et al., geodesic forehead height was significantly larger in girls than in boys in the present study. Two additional geodesic features—forehead width and upper lip height—were found to be significantly larger in boys than in girls in the current sample.

**Figure S1.**

The left of the flowchart describes the process in selecting sexually dimorphic facial features and training a gender classification model in Study 1 of Tan et al. (2017). The middle of the flowchart describes the steps taken in examining the generalisation of the model based on facial features of 40 boys and 40 girls with no known family history of ASD. The right of the flowchart describes the process in measuring the 11 facial features of 55 siblings and 129 controls were entered into the model for the computation of their facial masculinity scores.


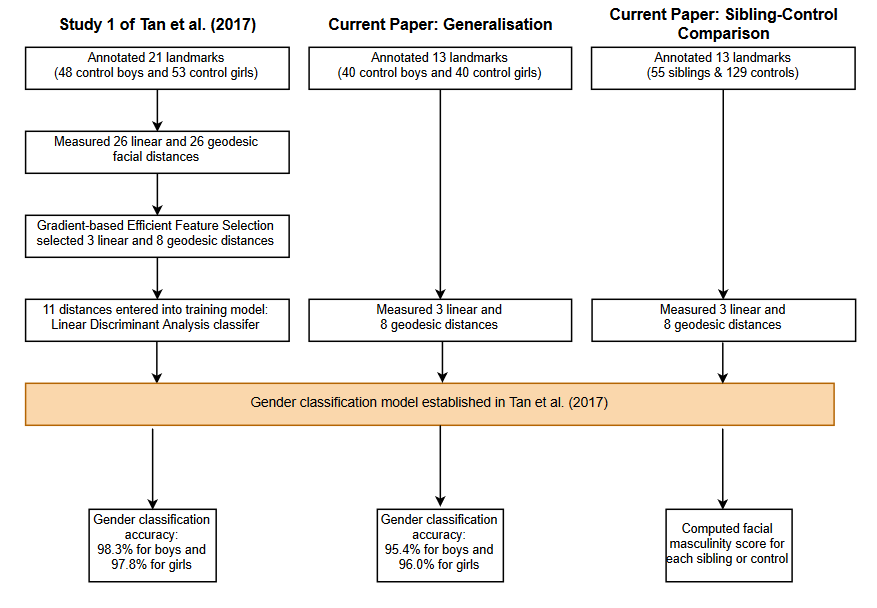

Supplement: Supplementary file 1 — Supplementary Material [file 41398_2020_695_MOESM1_ESM.docx]
